# Evaluation of tools to assess psychological distress: how to measure psychological stress reactions in citizen responders– a systematic review

**DOI:** 10.1186/s12873-019-0278-6

**Published:** 2019-11-04

**Authors:** Astrid Rolin Kragh, Fredrik Folke, Linn Andelius, Emma Slebsager Ries, Rasmus Vedby Rasmussen, Carolina Malta Hansen

**Affiliations:** 1 Emergency Medical Services Copenhagen, University of Copenhagen, Telegrafvej 5, DK-2750 Ballerup, Copenhagen, Denmark; 2 Department of Cardiology, Gentofte University Hospital, Gentofte Hospitalsvej 1, 2900 Hellerup Copenhagen, Denmark

**Keywords:** Citizen responders, Out-of-hospital cardiac arrest, Psychological impact, Stress disorder assessment

## Abstract

**Background:**

Dispatched citizen responders are increasingly involved in out-of-hospital cardiac arrest (OHCA) resuscitation which can lead to severe stress. It is unknown which psychological assessment tools are most appropriate to evaluate psychological distress in this population.

The aim of this systematic review was to identify and evaluate existing assessment tools used to measure psychological distress with emphasis on citizen responders who attempted resuscitation.

**Methods:**

A systematic literature search conducted by two reviewers was carried out in March 2018 and revised in July 2018. Four databases were searched: PubMed, PsycInfo, Scopus, and The Social Sciences Citation Index. A total of 504 studies examining assessment tools to measure psychological distress reactions after acute traumatic events were identified, and 9 fulfilled the inclusion criteria for further analysis. The selected studies were assessed for methodological quality using the Scottish Intercollegiate Guidelines Network.

**Results:**

The Impact of Event Scale (IES) and The Impact of Event Scale-Revised (IES-R) were the preferred assessment tools, and were used on diverse populations exposed to various traumatic events. One study included lay rescuers performing bystander cardiopulmonary resuscitation and this study used the IES. The IES and the IES-R also have proven a high validity in various other populations. The Clinical administered PTSD scale (CAPS) was applied in two studies. Though the CAPS is comparable to both the IES-R and the IES, the CAPS assess PTSD symptoms in general and not in relation to a specific experienced event, which makes the scale less suitable when measuring stress due to a specific resuscitation attempt.

**Conclusions:**

The IES and the IES-R seem to be solid measures for psychological distress among people experiencing an acute psychological traumatic event. However, only one study has assessed psychological distress among citizen responders in OHCA for which the IES-R scale was used, and therefore, further research on this topic is warranted.

## Background

Bystander cardiopulmonary resuscitation (CPR) is crucial to improve chances of survival following out-of-hospital cardiac arrest (OHCA) and is a cornerstone in both the European and American resuscitation guidelines, [1, 2]. Each year more than 350,000 OHCAs in the United States and 300,000 OHCAs in Europe are registered, [3, 4]. Several initiatives have been implemented in the past decades to increase bystander CPR, such as widespread campaigns to raise awareness among the general population, mandatory CPR training in schools, and other educational institutions, [5–7].

Consequently, the number of persons attempting CPR has increased in several countries, and in the U.S., rates of bystander CPR increased from 28% in 2005 to more than 46% in 2016, [8]. In Denmark the proportion of patients receiving bystander CPR rose from 21% in 2001 to 45% in 2010, [5].

The Cardiac Arrest Registry to Enhance Survival from the U.S. reported 30,063 cases had received bystander CPR in 2017. The number of bystanders involved in cardiac arrests is probably higher, since in many cases, several bystanders attempt resuscitation together. This number is likely to grow as authorities as well as international guidelines on resuscitation increasingly encourage citizens to attempt CPR and a growing number of countries have initiated citizen first responder programmes based on a smartphone application for dispatch of citizen responders to OHCAs, [1, 2, 9–11].

A number of qualitative interview studies have shown that citizen responders experience stress-related symptoms after participating in resuscitation attempts, [12–15]. However, the persistence and degree of the stress reactions among citizens who perform bystander CPR remains unknown. Understanding how to measure stress among bystanders who performed resuscitation is of utmost importance as this is not only a growing population, but also one acting by recommendation of health authorities.

The objective of this systematic review was to identify and evaluate existing assessment tools used to measure stress among people experiencing a traumatic event and evaluate whether these assessment tools are suitable for measuring psychological distress among citizen responders who have performed bystander CPR. The study aimed to identify one scale that relates to a specific traumatic event, is able to predict the risk of persistent psychological distress at 4 weeks after the event and has been validated among different population groups.

## Methods

This systematic review was reported using PRISMA guidelines (Preferred Reporting Items for Systematic Reviews and Meta-Analyses). The guidelines are preferred for reporting items for systematic reviews and meta-analyses and developed by Cochrane Collaboration, [16].

### Information sources and search

A literature review with a systematic search approved by a professional research librarian was conducted by two reviewers (AK, RR) via the University of Copenhagen between 17th and 20th of March 2018. The search was revised on 23th of July 2018. Studies were identified through searches in four databases using keywords combined from the PICO model (Table 1): PubMed, PsycINFO, Scopus, and The Social Sciences Citation Index. Covidence software program was applied to identify duplicates, screen-imported studies, and support the study selection process. This study was conducted without a review protocol.

**Table 1:** Search terms used in PICO model

Participant	Intervention/Phenomenon of Interest	Context or Outcome
Lay rescuer OR rescuer*	Psychological assessment tool	Psychological impact
Bystander rescuer OR bystander*	Stress disorder scale	Stress disorder
Persons who perceived traumatic event	Measure of distress	Post-traumatic stress symptoms
Persons who experiences traumatic event	Impact of event scale	Stress response syndromes

*Is a truncation symbol

### Eligibility criteria

The research question was developed following the PICOS (population, intervention, comparison, outcome and study design) format, [17]. Studies were considered eligible for inclusion if they met all of the following criteria: 1) Included citizen responders participating in a resuscitation attempt, or other persons involved in an acute psychologically traumatic experience (=population), 2) Applied or examined validated inventory tools to assess psychological distress or psychological impact after an experienced traumatic event (=intervention), 3) Measured stress disorders, psychological impact, or post-traumatic stress disorder (PTSD) related symptoms (=outcome). Excluded were studies with either of the following criteria: Performed on a population not experiencing an acute traumatic event, included persons under the age of 18 years or persons with a psychiatric diagnosis, and studies applying an inventory tool which was not validated. Conference abstracts and expert narratives were excluded due to the inability to evaluate the risk of bias. No language or time limitations were applied. Since many of the assessment tools for identifying stress disorders are based on questionnaires and interviews, both quantitative and qualitative studies were considered for inclusion.

### Study selection

Two reviewers (AR, RR) independently conducted the literature search based on the title and abstract and undertook the full-text review. In case of disagreement, the paper was re-read and discussed between the reviewers until consensus was reached. Studies that assessed psychological impact or stress disorders with a validated tool among people experiencing a specific acute traumatic event were selected.

Due to the lack of studies examining inventory tools to measure psychological stress reactions among citizen responders attempting resuscitation, there was considerable variability in the population group and therefore also in the cause of stress disorders in the included studies. Thus, as presented in Table 2, the selected studies included a wide range of participants (health care professionals, trauma patients, persons experiencing parental bereavement, citizen responders, etc.). The traumatic events varied from accidents to work-related trauma and injuries.

**Table 2:** Characteristics of all papers included in the review

Publication	Aim	Participants	Event	Assessment tool	Outcome
Zilberg et al., 1982	A validation of Impact of Event Scale	Patients who lost a parent	Experiences parental bereavement	IES	Stress response syndromes
Mooren et al., 2004	To assess the psychometric value of the Dutch version of the IES	Multiple	Work-related trauma, war-related trauma, disasters	IES	Post- traumatic stress responses
Beck et al., 2008	Factor structure, psychometric features and ability of IES- R to differentiate individuals w/wo PTSD	Motor vehicle accident suvivors	Motor vehicle accident involving actual or threatened death or serious injury	IES-R and CAPS	PTSD
Eid et al., 2009	Reliability and validity of the Norwegian version of IES-R	Psychology students (non-clinical sample)	Indirectly exposed through media reports from a tsunami disaster	IES-R	Post- traumatic stress symptoms
Sveen et al., 2010	Assess the Swedish version of IES and IES-R.	Patients with burns	Burn injury	IES-R and IES	PTSD
Bryant et al., 2011	To assess the capacity of acute stress disorder to predict posttraumatic psychiatric disorders	Trauma patients admitted to hospital after traumatic injury	Traumatic injury such as traumatic fall	Clinical Administered PTSD Scale (CAPS)	Acute stress disorder
Schütte et al., 2012	To examine the predictive variables of PTSD	Police Officers	Experienced traumatic incidence on duty	Structured Clinical Interview for DSM-IV and IES-R	PTSD and Acute Stress Disorder
Waller et al., 2015	Association between number of stressful events and severity of PTSD symptoms.	Australian Defence Force	Stressful events or traumatic experiences on deployment, workplace stressors and relationship/family issues	PCL-C	PTSD
Zijlstra et al., 2015	PTSD-related symptoms among lay rescuers performing CPR	First arriving lay rescuers performing bystander CPR	Participation in resuscitation attempt either by providing CPR or using AED	IES	Post- traumatic stress related symptoms

### Quality assessment

The risk of bias was assessed using The Scottish Intercollegiate Guidelines Network (SIGN) in each study, [18]. Sample strategy, method, and psychological assessment tool were evaluated, and each study was rated with -, + or ++. Specifications are presented in Table 3.

**Table 3:** Specifications of quality assessments

Publication	Quality (SIGN)	SIGN comments
Zilberg et al., 1982	–	Poorly described method
Mooren et al., 2004	++	Well described method and statistical analysis
Beck et al., 2008	++	Well described sample selection, randomization and method
Eid et al., 2009	+	Well described sample, poorly described method
Sveen et al., 2010	+	Inadequate described method
Bryant et al., 2011	+	Well described method
Schütte et al., 2012	+	Prospective study design
Waller et al., 2015	+	Retrospective design, but well described method
Zijlstra et al., 2015	++	Prospective study design

## Results

**Fig. 1:**
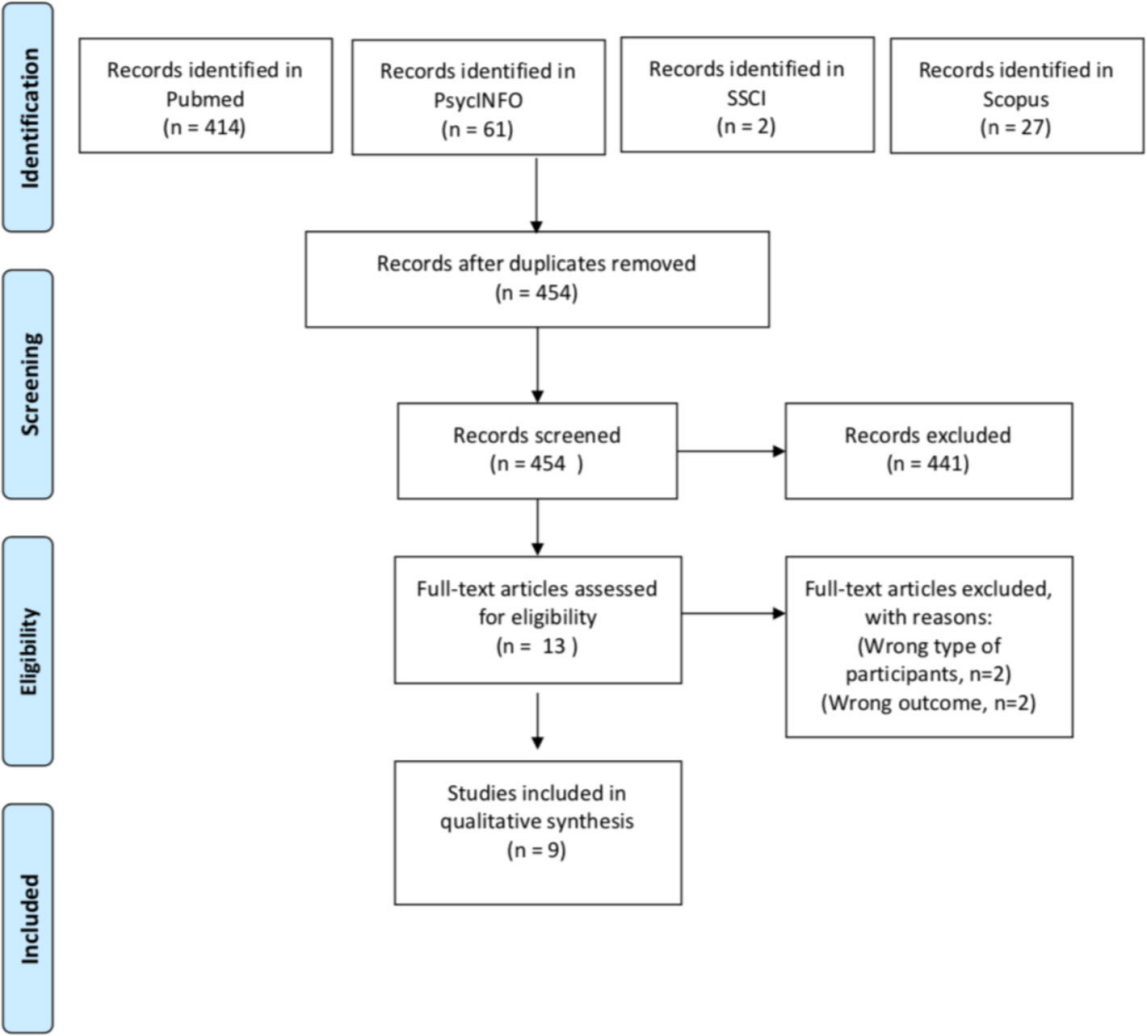
PRISMA flow diagram

The literature selection process and electronic search strategy is presented in the flow diagram Fig. 1 and resulted in the following: PubMed (*n* = 414), PsycINFO (*n* = 61), Scopus (*n* = 27), SSCI (*n* = 2). There were 50 duplicates identified, which resulted in 454 exclusive titles reviewed by AR and RR.

A total number of 441 studies were excluded as they did not meet the inclusion criteria described previously.

Of 13 papers that appeared relevant for inclusion, four were excluded following full-text reading resulting in a total number of nine studies included in a qualitative synthesis.

Four inventory tools were identified through the nine included studies: The Impact of Event Scale (IES), the Impact of Event Scale-Revised (IES-R), the Clinical Administered PTSD Scale (CAPS), and the Post-Traumatic Stress Disorder Checklist (PCL), which are briefly described in the following. All these tools have been validated on a wide range of populations such as police officers, burn patients and motor vehicle accident survivors, [19–21].

### The impact of event scale and the impact of event scale-revised

The Impact of Event Scale (IES) and Impact of Event Scale-Revised (IES-R) were most frequently used on different populations exposed to various acute traumatic events (*n* = 7). Both the IES and the IES-R have previously been validated and proven useful to predict PTSD, [19, 22, 23].

The original IES is a 15-item self-reported scale that assesses subjective distress caused by traumatic events, such as a resuscitation attempt. The scale is designed to assess the frequency of intrusive and avoidant stress symptoms with respect to a certain identified trauma, not related with traumatic symptoms in general. Intrusive symptoms refer to flashbacks to the event or dissociative reactions where it seems as if the trauma is reoccurring. Avoidant stress symptoms are expressed in questions that concern avoiding getting upset and trying not to think of the event.

The scale has been revised to the IES-R which contains seven additional items related to the hyperarousal symptoms of PTSD, such as anxiety and insomnia. These items were added to match the diagnose criteria for PTSD in the Structured Clinical Interview for Diagnostic and Statistical Manual of Mental Disorders (DSM). It has been shown that high IES scores 1 week after a traumatic event predicts PTSD 6 months later with 92% sensitivity [24, 25].

The maximum overall score possible in the IES-R is 12. The sum of the means of each subscale is recommended instead of raw sums. High levels of internal consistency and discriminative validity have been previously reported for the IES-R. A Japanese translation of the scale reported test-retest values and Rash et al. described a high level of internal consistency among total and subscale scores (Crohnbach’s alpha 0.95). The convergent validity has been reported with consistent and high correlations between the IES-R and related measures of PTSD [20, 26–28].

A prospective observational study conducted by Zijlstra et al., evaluated the perceived short-term impact on psychological wellbeing of lay rescuers performing bystander CPR, [29]. Furthermore, they aimed to investigate the level of PTSD-related symptoms among bystanders 4–6 weeks after resuscitation attempts. This study was the only publication from the literature search measuring stress reactions with a validated inventory tool among citizen responders attempting resuscitation. The authors applied the IES to measure psychological symptoms of PTSD among the participants (*n* = 189), by sending the scale to citizen responders 4 weeks after the resuscitation attempt. The authors found that 41% of the responders reported no/mild short-term psychological impact, 46% bearable impact and 24% severe impact on the IES. However, no of the citizen responders scored > 26 (moderate or severe stress), 19% scored 9–25 (mild stress), and 81% scored 0–8 (no stress). None of the citizen responders reporting severe or bearable psychological impact on the short term scored more than mild stress levels on the IES (4–6 weeks after the event).

The IES has furthermore been validated to assess the psychological impact of a variety of traumas in a study by Van der Ploeg et al., [23]. In this study, the authors evaluated the psychometric value of the Dutch version of the IES in three different samples of individuals who had experienced various traumatic stressors (work-related trauma, war-related trauma, and disasters). The authors found that the IES is a justified and valid instrument with a robust factor structure. An earlier study by Zilberg et al. reported a detailed description of the IES and encouraged cross-validational data and analyses, [30]. The study concluded that the IES is a sensitive measure of change, suitable for intervention studies utilizing repeated measurements over time.

The IES-R has correspondingly to the IES been validated on a variety of different populations experiencing acute psychologically traumatic events. In a publication by Sveen et al., the authors studied the Swedish version of the IES-R and validated it against the DSM in a population of burn victims, [19]. The authors examined the ability of the scale to discriminate between individuals with and without PTSD. They found that participants with a positive PTSD diagnosis had higher scores on IES-R than those without a diagnosis. The results showed that the IES-R has good properties as a screening tool for the diagnosis of PTSD in patients with burns 1 year after injury. The IES-R has also proven useful in a study by Beck et al. based on a sample of motor vehicle accident survivors (*n* = 182) who sought treatment for mental health problems following the injury, [20]. The authors examined the factor structure of the IES-R and its related psychometric features as well as the ability of the scale to differentiate between individuals with and without diagnosable PTSD. Based on their results, the authors suggested that the IES-R is not simply a measure of general distress but appears to have specific agreement in the assessment of PTSD symptomatology. The authors concluded that the scale had possibility to differentiate between individuals with and without PTSD although it was not developed as a diagnostic tool. The study suggests that the IES-R seems to be a solid measure of post-trauma phenomena that can enlarge related assessment approaches.

Another prospective study by Schütte et al. provides evidence that the IES-R is a suitable scale in predicting the development of stress symptoms and PTSD after experiencing an acute psychologically traumatic event, [21]. The authors investigated the predictors for the development of PTSD in 59 police officers who had experienced a traumatic incident during duty where the participants completed the IES-R immediately after the event and 6 months later.

The reliability and validity of the IES-R has moreover been examined in a Norwegian study by Eid et al., [31]. The study was based on a non-clinical sample consisting of 311 undergraduate psychology students who were asked to review media reports from a tsunami disaster and were subsequently asked to rate their traumatic symptoms. The authors found that the Norwegian version of the IES-R has satisfactory psychometric properties with good reliability an accuracy in terms of detecting dimensions of PTSD-symptoms. The authors recommended to use the scale to measure traumatic symptoms with respect to a certain trauma in future studies. However, none of the students had been personally exposed to the tsunami but were only exposed through the media. The IES-R was administered to the students about 3 weeks after the disaster.

### The CAPS - clinical administered PTSD scale

The Clinical administered PTSD scale (CAPS) is known as the golden standard in PTSD assessment and has been revised several times. The scale is a 30-item structured clinical interview that in addition to the 17 PTSD symptoms includes items pertaining to the dissociative symptoms required for a diagnosis of Acute Stress Disorder. The newest version of the scale (CAPS-5) is a structured interview that assesses PTSD symptoms over the past week and makes current as well as lifetime diagnosis of PTSD, [32].

A longitudinal study by Bryant et al. was performed among survivors of traumatic injury, [33]. The study reported analyses that evaluated the capacity of Acute Stress Disorder in the initial month after trauma to predict a range of disorders including PTSD 12 months later. Acute Stress Disorder was assessed using the CAPS in the initial month after trauma. The authors found that 10% of the participants met criteria for Acute Stress Disorder at the initial assessment. After 12 months, 31% had a psychiatric disorder of which 10% met the criteria for PTSD. The study concluded that the majority of the trauma survivors who developed PTSD did not meet Acute Stress Disorder criteria in the initial month. Only a third of patients who did meet Acute Stress Disorder criteria developed PTSD. The acute stress disorder criteria measured by the CAPS has limited utility in identifying trauma-exposed individuals who are at risk for PTSD.

### The PCL – post-traumatic stress disorder checklist

The PCL is a psychometrically validated checklist developed to measure the 20 DSM-5 symptoms of PTSD. The scale is a self-reported measure and has various purposes, such as PTSD-screening, PTSD-diagnosing, and symptom change monitoring during and after treatment, [34, 35]. A retrospective cohort study conducted by Waller et al. investigated the association between numbers of stressful events and the severity of PTSD symptoms using the PCL. The authors aimed to explore if new stressful events trigger memories of previous events. A cohort consisting of 1119 Australian military personnel deployed to the Middle East, was asked to report traumatic exposures associated with deployment. Scores on the PCL and stressful events were measured. The study found that personnel reporting more events had a higher mean PLC-score compared to those who reported no events. The study concluded that number of stressful events was significantly associated with more symptoms of PTSD, [36].

## Discussion

This systematic review presents an overview over existing assessment tools used to measure psychological distress with emphasis on stress reactions among citizen responders who attempted resuscitation. We found that both the IES and the IES-R are solid measures for psychological distress among people experiencing an acute psychological traumatic event, but only one study has assessed psychological distress among citizen responders after participation in resuscitation attempts using the IES score. Therefore, future studies examining psychological distress among citizen responders in out-of-hospital cardiac arrest are warranted.

The majority of the studies included in this review (seven out of nine) examined the IES or IES-R and only one study, [29] applied a validated tool (the IES) to measure psychological stress in citizen responders who attempted resuscitation in OHCA. In accordance with the study provided by Van der Ploeg et al., [23] who suggested a predictive validity of PTSD-symptoms at minimum 4 weeks after event, Zijlstra et al. sent the questionnaire by e-mail 1 month after the resuscitation attempt, which suggests results are valid and reliable.

Despite this, a couple of limitations are worth mentioning. Several of the participants (42%) in the study by Zijlstra et al. were off-duty professional rescuers who might cope better with stress than lay rescuers. According to a study using the IES examining people who experienced an avalanche disaster, volunteers meet PTSD criteria significantly more often than professional rescuers, [37]. Another limitation is that an interview was conducted with all bystanders included in the study before they answered the questionnaire. A recent qualitative study found that debriefing bystanders stimulates the ability to cope with emotional reactions, [38]. Consequently, the interview may have served as debriefing and thus influenced the stress score and biased the results. Moreover, the study did not include measurement of hyperarousal symptoms since the authors applied the original version of the IES and not the revised version. The study did not investigate the persistence of the perceived stress among the citizen responders as no long-term follow-up was performed.

Overall, the study contributes with important knowledge of clinical relevance to measure psychological distress among citizen responders participating in resuscitation attempts.

We found both the IES and the IES-R useful to predict PTSD-related symptoms in persons experiencing an acute traumatic event comparable to a resuscitation attempt. Since both scales have been validated on various groups comparable to persons attempting resuscitation, the scales might serve useful on citizen responders as well.

One study applied the CAPS to measure psychological distress among survivors of traumatic injury, [33]. A review investigating the present literature within CAPS established that the scale in general matches the results for self-report PTSD measures - particularly the IES, [39]. However, CAPS is a structured interview specifically designed for detecting PTSD. The scale assesses PTSD symptoms in general, and not in relation to a specific experienced traumatic acute event. This makes the scale unsuitable for use on citizen responders participating in resuscitation attempts. Another important issue is the inability to predict PTSD based on the presence of stress symptoms at the initial month after event since the majority of the trauma survivors who developed PTSD did not meet the Acute Stress Disorder criteria in the initial month. Since our review seeks to identify a scale with the ability to distinguish citizen responders in risk of developing stress disorders at 4 weeks post event, the CAPS does not seem to be useful as a measure. The Acute Stress Disorder Scale did not seem appropriate as a screening instrument to predict PTSD since this scale is not suitable to measure persistent psychological distress and has only been validated in few studies.

Some studies indicate that experiencing multiple similar traumatic events increases the risk of a high score on the IES-R. The study conducted by Waller et al. proved a significant association between numbers of experienced traumatic events and severity of PTSD symptoms, [36]. The findings are consistent with the cumulative effects of stressful events being associated with increasing PTSD symptoms, [40]. Hence, it might be necessary to consider the presence of earlier traumatic events among citizen responders performing CPR, to predict the persons in risk of developing PTSD related symptoms. This information may be valuable if citizen responders are exposed to more than one resuscitation attempt and may be of particular importance in programs with dispatch registered citizen responders since these have a higher chance of providing CPR in multiple cardiac arrests compared to the general population. Additionally, the studies included in this review suggest the IES/IES-R may be used in repeated assessments to predict the long-term consequences of a traumatic event such as resuscitation attempts. Zilberg et al. reported that the IES is suitable for intervention studies utilizing repeated measurements over time, [30]. This is in accordance with the study by Van der Ploeg et al. who applied the scale 1 month after the event, and found the scale to be a valid instrument with a robust factor structure, [23]. However, the study did not provide a lack of a comparison group with a clinical interview, which is typically done as part of scale validation. To achieve reliability and to determine the development of stress over time, it may be necessary to measure stress a short time after injury (30 days) and repeat measurement after 1 year. The study provided by Eid et al. found a significantly higher severity of PTSD-related symptoms among women than men, [31]. A review of the epidemiologic literature on stress disorders found the prevalence of PTSD estimated to be 5% among men and 10% among women, [41]. The presence of higher severity of PTSD-related symptoms among women than men is needed to have in mind when measuring psychological distress among citizen responders.

## Limitations

This systematic literature review has several limitations. Only one study applying a validated psychological assessment tool to measure stress among citizen responders to OHCA was identified and thus, limited knowledge is available to address the aim of the study. As for any systematic literature review, there is a risk of publication bias since the searches were limited to published articles. The lack of studies using validated inventory tools to measure stress among citizen responders who performed CPR made it necessary to expand the search to participants experiencing a more undefined acute traumatic event and attempting CPR at cardiac arrest might elicit different psychological reactions compared to those who were physically injured. Although all included studies are observational, both SIGN and PRISMA guidelines were used. Further studies are needed regarding a broader sample of bystanders who attempted CPR.

## Conclusion

Both the IES-R and the IES seem to be solid measures for psychological distress among people experiencing an acute traumatic event, although there is a lack of evidence within the field of citizen responder resuscitation. Further studies validating the scales as inventory tools for measuring psychological distress among citizen responders are recommended.

## Data Availability

Data sharing is not applicable to this article as no datasets were generated or analysed during the current study.

## Abbreviations

CAPS: The Clinical administered PTSD scale
CPR: Bystander cardiopulmonary resuscitation
DSM: Diagnostic and Statistical Manual of Mental Disorders
IES: The Impact of Event Scale
IES-R: The Impact of Event Scale Revised
OHCA: Out-of-hospital cardiac arrest
PCL: Post-traumatic stress disorder checklist
PRISMA: Preferred Reporting Items for Systematic Reviews and Meta-Analyses
PTSD: Post-traumatic stress disorder
SIGN: The Scottish Intercollegiate Guidelines Network
